# Dr. Henry Hodgen Mudd

**Published:** 1900-01

**Authors:** 


Dr. Henry Hodgen Mudd, of St. Louis, Mo., died at his residence in that city November 20, 1899, aged 55 years. Since the death of his uncle, Dr. John T. Hodgen, Dr. Mudd has been recognised as the leading surgeon in the southwest, and his loss is great to St. Louis and vicinity.

At the time of his death he was dean of the medical department of Washington University and professor of surgery in that institution. He is survived by his father, two brothers and four children.

